# A prospective study on prognostic value of ventilatory efficiency in asymptomatic patients with severe primary mitral regurgitation

**DOI:** 10.1371/journal.pone.0326418

**Published:** 2025-07-09

**Authors:** Johannes Bergsten, Tomasz Baron, Eva-Maria Hedin, Nermin Hadziosmanovic, Andrei Malinovschi, Frank A. Flachskampf

**Affiliations:** 1 Department of Heart and Lung Diseases, Uppsala University Hospital, Uppsala, Sweden; 2 Department of Medical Sciences, Uppsala University, Uppsala, Sweden; 3 Uppsala Clinical Research Centre, Uppsala University, Uppsala, Sweden; 4 Department of Surgical Sciences, Uppsala University, Uppsala, Sweden; Charité Universitätsmedizin Berlin - Campus Virchow-Klinikum: Charite Universitatsmedizin Berlin - Campus Virchow-Klinikum, GERMANY

## Abstract

**Objectives:**

Patients with severe primary mitral regurgitation (PMR) remain asymptomatic at first. In the long term, however, severe PMR leads to cardiac decompensation. Exercise testing in asymptomatic PMR is recommended in selected patients by guidelines. Cardiopulmonary exercise testing (CPET), which additionally measures ventilation (VE), oxygen consumption (VO_2_) and carbon dioxide production (VCO_2_), has been scarcely studied in PMR. We hypothesized that CPET might have prognostic value in asymptomatic PMR and therefore studied if CPET, including assessment of ventilation efficiency, has prognostic value for asymptomatic patients with severe PMR.

**Methods:**

Asymptomatic patients with severe PMR were prospectively recruited between 2013 and 2018. Exclusion criteria were coronary artery disease, chronic kidney disease, diabetes mellitus, concomitant valve disease, symptomatic lung disease or class 1 recommendation for valvular surgery. Echocardiography and serial CPET were conducted at one university hospital in Sweden. Primary outcome was mitral valve intervention.

**Results:**

Forty-eight patients were recruited to the study. Median follow-up period was 4.4 (2.1–6.9) years, during which 28 (58%) patients underwent mitral valve surgery. Ventilation efficiency, the relationship of VE to VCO_2_ during CPET, predicted surgical treatment of the mitral valve. Increased VE/VCO_2_ ratio at the anaerobic threshold had the highest predictive value, remaining an independent predictor after adjusting for impaired VO_2_ at peak exercise (HR 4.42 (1.52–12.92), p = 0.007) and echocardiographic thresholds for left ventricular and atrial remodelling, as defined by current guideline-based recommendation for intervention (HR 3.72 (1.41–9.82), p = 0.008).

**Conclusion:**

Impaired ventilatory efficiency, but not peak VO_2_, predicted surgical treatment of the mitral valve in asymptomatic patients with severe PMR. Ventilatory efficiency, a CPET index less dependent on peak exercise performance, may be a new useful tool in risk stratification.

**Key Message**

Little is known about CPET in PMR. The few existing studies focus on the prognostic value of peak VO2. We investigated the prognostic value of CPET in asymptomatic patients with severe PMR. Impaired ventilation efficiency, the relationship of VE to VCO2, predicted surgical treatment of the mitral valve. Ventilatory efficiency may be a useful tool for risk stratification of asymptomatic patients with severe PMR.

## Introduction

Mitral regurgitation causes the left ventricle (LV) to eject volume in systole both forward through the aortic valve and backward through the regurgitant mitral valve. In severe mitral regurgitation, the end-diastolic left ventricular volume (LVEDV) progressively increases to maintain cardiac output. In the short term, patients typically remain asymptomatic. In long-term, however, reduced LV contractility, reduced left ventricular ejection fraction (LVEF), elevated left atrial (LA) pressure and pulmonary hypertension ensue with cardiac decompensation. Guidelines recommend intervention depending on heart failure symptoms and/or objective signs of LV and LA remodelling, and volume overload, which typically are evaluated by cardiac imaging, usually echocardiography and in some cases also cardiovascular magnetic resonance [1,2].

Mitral regurgitation is classified as primary (valve-related) or functional/secondary (due to LV and/or LA dilatation). Primary mitral regurgitation (PMR) is mostly due to degenerative mitral valve disease with mitral valve prolapse. Prolapse may be due to fibroelastic deficiency, mostly involving the P2 segment of the valve, or represent more extensive leaflet tissue thickening with bileaflet prolapse, suggesting Barlow’s disease [3].

Assessing the onset of symptom in PMR can be challenging in clinical practice, and exercise testing is recommended to detect subclinical functional impairment in selected patients by guidelines of the European Society of Cardiology and American College of Cardiology [1,2]. Cardiopulmonary exercise testing (CPET), which in addition to conventional exercise testing measures ventilation (VE), oxygen consumption (VO_2_), and carbon dioxide production (VCO_2_), has not been used extensively to assess patients with valvular heart disease [4]. VO_2_ at peak exercise has been the gold standard for assessing cardiopulmonary limits with CPET. More recently, ventilatory efficiency has gathered interest for the assessment of non-valve related heart failure [5–7].

Current clinical recommendations for CPET data assessment proposes incorporating both the Weber [8] and Ventilatory Classification System [9] for prognostic stratification in patients with non-valvular heart failure, using peak VO₂ (Class A: > 20 mL/kg/min; Class B: 16–20 mL/kg/min; Class C: 10–16 mL/kg/min; Class D: ≤ 10 mL/kg/min) and VE/VCO₂ slope (Class I: ≤ 29.9; Class II: 30–35.9; Class III: 36–44.9; Class IV: ≥ 45), respectively [10]. Similarly, a consensus statement has highlighted the diagnostic and prognostic value of both peak VO₂ and VE/VCO₂ slope, in patients with heart failure with preserved ejection fraction [11]. In addition to static cut-off values for peak VO₂ and VE/VCO₂ slope, both position papers acknowledge the relevance of age- and sex-specific reference values.

Despite its potential utility, CPET remains underutilized in the assessment of valvular heart disease [4], and evidence regarding its prognostic significance in PMR remains limited. In this study the goal was to investigate the prognostic value of CPET, including ventilation efficiency, in asymptomatic patients with severe PMR.

## Methods

### Study group

Asymptomatic patients with chronic severe PMR were recruited to the study between 14 October 2013 and 6 March 2018. Exclusion criteria were history of coronary artery disease, chronic kidney disease, diabetes mellitus, non-sinus rhythm, LV dysfunction, pulmonary hypertension, concomitant moderate or severe valve disease, symptomatic lung disease or class 1 guideline recommendation for valvular intervention. All patients were included and evaluated at the Department of Cardiology and Clinical Physiology, Uppsala University Hospital, and re-evaluated after one year. Later follow-up was conducted by reviewing medical records. All medical decisions were at the discretion of the treating physician and involved heart team. The study was approved by the Regional Ethical Review Board at Uppsala University (Dnr 2012/543). Patients provided written informed consent.

### Echocardiography

Transthoracic echocardiography (Vivid-9, GE Healthcare, Stockholm, Sweden) was performed on the same day as the CPET by an experienced sonographer, according to guidelines [12]. Analyses (EchoPac version 203, GE Healthcare) were performed according to current recommendations by a single experienced physician.

The severity of mitral regurgitation was assessed using an integrative, guideline-endorsed approach incorporating multiple echocardiographic parameters, including mitral valve morphology, colour Doppler assessment of the regurgitant jet, proximal flow convergence zone, continuous-wave Doppler profile of the regurgitant jet, vena contracta width, pulmonary vein flow pattern, mitral inflow profile, effective regurgitant orifice area, regurgitant volume, and LV–LA remodelling, in accordance with current echocardiographic recommendations [13].

LA volume was calculated with the biplane method. LV volumes and LVEF were assessed using the Biplane Modified Simpson’s Method [14]. The right ventricular systole pressure (RVSP) was estimated from the tricuspid regurgitant velocity, using the simplified Bernoulli equation (RVSP = 4V^2^ + right atrial pressure estimated from vena cava inferior size and collapsibility) [15].

### Cardiopulmonary exercise testing

CPET was performed in semi-supine position with bicycle ergometer (JaegerOxycon Pro, Erich Jaeger GmbH, Hoechberg, Germany). The device was calibrated before each examination according to manufacturer’s recommendation. Medications were not stopped before the exercise testing. A continuous 12-lead electrocardiogram was used. The blood pressure was monitored every two minutes.

A period of 2–3 minutes of resting on bicycle CPET data collection was followed by an exercise phase, with no prior unloaded cycling. For all patients, the incremental exercise phase started at 50 watts with a minute-by-minute increment of 10 watts. A cadence of 60 rpm was maintained. The patients performed a symptom-limiting exercise, to a self-estimate grade of 17 of 20, on the Borg Rating of Perceived Exertion scale [16]. The peak exercise workload was compared with Swedish reference values [17].

### CPET data acquisition and analysis

The VE, VO_2_ and VCO_2_ were reported as average values every 15 seconds. The respiratory exchange ratio (RER) was calculated as the ratio of VCO_2_ to VO_2_. The anaerobic threshold was defined as when RER reached 1.0 during incremental exercise. The O_2_-pulse was calculated by dividing VO_2_ by heart rate at peak exercise. The VE/VCO_2_ slope was calculated from rest to the anaerobic threshold. VE/VCO_2_ ratios were analysed at the anaerobic threshold and at its lowest value.

### Comparison to reference values and criteria for defining normal CPET parameters

CPET indices were primarily compared against reference values reported by Gläser et al. [18] that are clinically used in Sweden. As normal values for the VE/VCO_2_ ratio at its lowest value were not reported by Gläser et al., this parameter was calculated as a 90-second average following the method established by Sun et al. [19], and compared with the corresponding reference values. Normal reference ranges were defined using the lower limit of normal (5th percentile) and upper limit of normal (95th percentile). Concomitant impaired peak VO_2_ and reduced O_2_-pulse was interpreted as signs of cardiac limitation. A ventilatory limitation was defined as concomitant impaired peak VO_2_ and a reduced ventilatory reserve < 15% of maximal voluntary ventilation, estimated by multiplying the forced expiratory volume in 1 second (FEV_1_) by 40 [20]. FEV_1_ was compared with reference values proposed by Hedenström et al. [21,22].

### Statistical analysis

Continuous variables are presented as mean ± standard deviation or as medians and interquartile ranges. Categorical variables are presented as the number (percentage) of patients with the characteristic. For all analyses, two-sided P-values less than 0.05 were defined as significant. Group comparisons were performed using Mann-Whitney test, Fisher’s test and for paired groups Wilcoxon test. Cox-proportional hazards regression models were used to determine variable association with surgical treatment of the mitral valve. The underlying proportional hazards assumptions of the Cox proportional hazards models were veriﬁed by visual inspection of the Schoenfeld residuals. Time related valve surgeries were plotted with Kaplan-Meier curves and compared with log-rank test. Statistical analyses were performed using GraphPad Prism version 10.2.3 for macOS (GraphPad Software, La Jolla, CA, USA) and SAS software version 9.4 (SAS Institute, Cary, North Carolina).

### Patient and public involvement statement

Research question and outcome measures were defined by the authors. Patients’ symptoms however were of paramount importance, as stated in the description of recruitment. Patients were recruited by the criteria stated in the Methods section and were identified as candidates from the workflow of the echocardiography laboratory and the cardiology ambulance. They were not involved in further recruitment or conduct of the study. Some, but not all, patients return to regular follow-up visits to our clinic. Since the results of the study have no direct impact on the long-term management of the disease, which anyway by now is in the majority post-surgery, the results are not routinely discussed with the patients. Patients or public were not involved in devising and conducting the study.

## Results

### Study group

Forty-eight asymptomatic patients with chronic severe PMR were recruited to the study (Table 1). Age at inclusion was 62.2 ± 9.7 years. A majority, 44 (92%) of the patients were male. A history of hypertension was present in 29 (60%) patients. NT-pro-BNP, glomerular filtration rate, haematocrit and Troponin I were measured. Biomarker levels did not differ between patients having a surgical treatment of the mitral valve or not during follow-up. Patient characteristics, medication and biomarkers are presented in Table 1.

**Table 1:** Clinical data including a comparison of patients with and without mitral valve surgery during follow-up.

	Total (n = 48)	No MV surgery (n = 19)	MV surgery (n = 29)	*p*
Age (years)	62.2 ± 9.7	63.4 ± 8.5	61.3 ± 10.5	0.53
Gender (male)	44 (92%)	18 (95%)	26 (90%)	1.00
Follow-up (years)	4.4 (2.1-6.9)	6.9 (6.4-8.0)	2.4 (1.2-3.5)	<0.001
BMI (kg/m^2^)	25.4 ± 2.8	25.1 ± 2.7	25.6 ± 2.8	0.78
BSA (m^2^)	2.00 ± 0.16	1.98 ± 0.20	2.01 ± 0.13	0.69
ECG, sinus rhythm	48 (100%)			
NYHA, class I	48 (100%)			
Smoker	0 (0%)			
Angina	0 (0%)			
Diabetes	0 (0%)			
Atrial fibrillation	0 (0%)			
Asthma	1 (2%)		1 (3%)	1.00
Hypertension	29 (60%)	12 (63%)	17 (59%)	1.00
**Medication**				
Acetylsalicylic acid	7 (15%)	3 (16%)	4 (14%)	1.00
beta-blockers	9 (19%)	2 (11%)	7 (24%)	0.29
RAAS inhibitor	25 (52%)	10 (53%)	15 (52%)	1.00
Calcium channel blockers	7 (15%)	4 (21%)	3 (10%)	0.41
Digoxin	2 (4%)	0 (0%)	2 (7%)	0.51
Spironolactone	1 (2%)	0 (0%)	1 (3%)	1.00
Hydrochlorothiazide	3 (6%)	1 (5%)	2 (7%)	1.00
**Biomarkers**				
NT-proBNP (ng/L)	149 ± 144	125 ± 91	164 ± 170	0.57
GFR (mL/min/1.73m^2^)	83 ± 13	85 ± 12	82 ± 13	0.44
Hematocrit (%)	42 ± 3.5	43 ± 2.8	42 ± 4.0	0.56
Troponin I (ng/L)	5.7 ± 8.8	4.7 ± 4.1	6.3 ± 10.9	0.42

Continuous variables are presented as mean ± standard deviation or as medians and interquartile ranges. Categorical variables are presented as the number (percentage) of patients with the characteristic. Abbreviations: MV, mitral valve; BMI, body mass index; BSA, body surface area; ECG, electrocardiogram; NYHA, New York Heart Association Classification; RAAS, renin-angiotensin-aldosterone system; GFR, glomerular filtration rate.

### Echocardiography

All patients were confirmed to have severe mitral regurgitation. The most common underlying pathology was isolated posterior mitral leaflet prolapse, observed in 38 patients (79%) (Table 2). The LV was dilated, with a LVEF > 60% in the majority, except for five individuals who had an LVEF ranging from 56–59%. The LVESD was < 40 mm in most cases, although four patients had an LVESD between 40–43 mm. The LA was dilated, with five individuals exhibiting a LA volume index > 60 mL/m². Tricuspid annular plane systolic excursion was 29 ± 4 mm, consistent with normal right ventricular systolic function. The estimated RVSP was within the normal range, with a mean value of 30 ± 6 mmHg; no patient presented values > 50 mmHg.

**Table 2:** Echocardiography data of patients with and without mitral valve surgery during follow-up.

	Total (n = 48)	No MV surgery (n = 19)	MV surgery (n = 29)	*p*
HR (beats per minute)	66 ± 8	68 ± 7	64 ± 8	0.95
SBP (mmHg)	139 ± 14	141 ± 14	138 ± 14	0.47
LVEF (%)	66 ± 4	66 ± 5	66 ± 4	0.53
GLS (%)	21 ± 3	21 ± 3	22 ± 3	0.79
LVEDD (mm)	55 ± 5	53 ± 6	57 ± 4	**0.008**
LVESD (mm)	34 ± 4	33 ± 4	35 ± 4	**0.02**
LVEDV (mL/m^2^)	97 ± 19	87 ± 14	104 ± 18	**0.004**
LAVI (mL/m^2^)	42 ± 20	35 ± 11	46 ± 24	0.052
TAPSE (mm)	29 ± 4	28 ± 4	29 ± 4	0.60
RVSP (mmHg)	30 ± 6	28 ± 3	32 ± 6	0.10
**Mitral valve**				
Prolapse	48 (100%)			
Posterior leaflet	38 (79%)	16 (84%)	22 (76%)	0.72
Anterior leaflet	3 (6%)	1 (5%)	2 (7%)	1.00
Both leaflets	7 (15%)	2 (11%)	5 (17%)	0.69
EROA (cm^2^)*	0.50 ± 0.39	0.37 ± 0.19	0.60 ± 0.46	**0.03**

Continuous variables are presented as mean ± standard deviation. Categorical variables are presented as number (percentage). Number of observations: 48 or 44 (*), as indicated.

Abbreviations: MV, mitral valve; HR, heart rate; SBP, systolic blood pressure; LVEF, left ventricular ejection fraction; GLS, global longitudinal strain; LVEDD, left ventricular end-diastolic diameter; LVESD, left ventricular end-systolic diameter; LVEDV, left ventricular end-diastolic volume; LAVI, left atrial volume index; TAPSE, tricuspid annular plane systolic excursion; RVSP, right ventricular systolic pressure; EROA, effective regurgitant orifice area.

### Cardiopulmonary exercise testing

The mean exercise duration was 8.9 ± 3.3 minutes, and the peak workload achieved was 136 ± 33 watts (Table 3). All patients were in sinus rhythm at rest and throughout exercise testing. FEV₁ was 87 ± 13% of predicted values, and the breathing reserve at peak exercise was 49 ± 13%, indicating no evidence of ventilatory limitation in any patient. Peak VO_2_ was 22.5 ± 5.5 mL/min/kg, there was no difference in VO₂ values between patients who subsequently underwent mitral valve surgery and those who did not.

**Table 3:** Results from CPET of patients with and without mitral valve surgery during follow-up.

	Total (n = 48)	No MV surgery (n = 19)	MV surgery (n = 29)	*p*
Workload (watts)	136 ± 33	132 ± 30	138 ± 35	0.52
Watts (% of predicted value)	70 ± 14	69 ± 12	71 ± 15	0.46
SBP (mmHg)	217 ± 24	215 ± 21	219 ± 26	0.50
HR (bpm)	131 ± 15	131 ± 17	130 ± 13	0.95
HR (% of age-predicted maximal HR)	83 ± 9	84 ± 11	82 ± 7	0.90
FEV_1_ (% of predicted value)	87 ± 13	85 ± 14	87 ± 13	0.68
RER	1.11 ± 0.09	1.13 ± 0.09	1.09 ± 0.09	0.21
Cardiac limitation	8 (17%)	4 (21%)	4 (14%)	0.70
**Oxygen consumption**				
VO_2_ @peak (mL/min/kg)	22.5 ± 5.5	21.6 ± 4.2	23.0 ± 6.3	0.81
VO_2_ @AT (mL/min/kg)	15.8 ± 3.1	15.5 ± 2.3	15.9 ± 3.6	0.81
VO_2_ @peak (impaired; < LLN, Gläser et al.)	13 (27%)	6 (32%)	7 (24%)	0.74
VO_2_ @AT (impaired; < LLN, Gläser et al.)	0 (0%)			
**O**_**2**_ **-pulse**				
O_2_-pulse (mL/min/bpm)	14.0 ± 3.2	13.3 ± 3.1	14.4 ± 3.2	0.31
O_2_-pulse (impaired; < LLN, Gläser et al.)	9 (19%)	4 (21%)	5 (17%)	1.00
**Ventilatory efficiency**				
VE/VCO_2_ @AT*	31.0 ± 3.5	30.6 ± 2.4	31.7 ± 4.1	0.35
VE/VCO_2_ @lowest value	30.1 ± 3.2	29.6 ± 1.9	30.5 ± 3.9	0.55
VE/VCO_2_ slope*	28.6 ± 3.6	27.9 ± 2.7	29.1 ± 4.1	0.31
VE/VCO_2_ @AT* (increased; > ULN, Gläser et al.)	10 (21%)	1 (6%)	9 (35%)	**0.03**
VE/VCO_2_ @lowest value (increased; > ULN, Sun et al.)	5 (11%)	0 (0%)	5 (19%)	0.07
VE/CO_2_ slope* (elevated; > ULN, Gläser)	5 (11%)	0 (0%)	5 (19%)	0.07

Continuous variables are presented as mean ± standard deviation, and categorical variables are expressed as number (percentage) of patients. Number of observations: 48 or 44 (*), as indicated.

Abbreviations: MV, mitral valve; SBP, systolic blood pressure; HR, heart rate; FEV₁, forced expiratory volume in one second; RER, respiratory exchange ratio; VO₂, oxygen consumption; AT, anaerobic threshold; VE, ventilation; VCO₂, carbon dioxide production; LLN, lower limit of normal; ULN, upper limit of normal.

A total of 44 patients (92%) reached the anaerobic threshold, permitting assessment of both the VE/VCO₂ slope and the VE/VCO₂ ratio at the anaerobic threshold. The VE/VCO₂ ratio at its lowest value was calculated for all 48 patients. Patients who subsequently underwent mitral valve surgery had a higher prevalence of an increased VE/VCO₂ ratio at its lowest value (9 (35%) versus 1 (6%), p = 0.03). Notably, a high VE/VCO₂ ratio at the anaerobic threshold, and an elevated VE/VCO₂ slope, were observed exclusively among patients who later underwent surgery (5 (19%) versus 0, p = 0.07 for both comparisons).

### One-year follow-up CPET

Forty-three (90%) patients participated in one-year follow-up CPET. At the time of one-year follow-up, mitral valve surgery had been conducted in 5 of 43 patients. CPET Δ-value calculations (1-year follow-up minus baseline values) were calculated for the non-operated 38 patients (S1 Table A). Because of partial data corruption, ventilatory values, except peak VO_2_, were not recorded for 12 of 38 (32%) patients. The remaining 26 of 38 (68%) patients had complete data. CPET Δ-values did not differ between patients who had a surgical treatment of the mitral valve or not, during the continued follow-up period.

### Clinical outcome

Median follow-up was 4.4 (2.1–6.9) years. In total 28 (58%) patients underwent mitral valve intervention. Indications for surgery was onset of symptoms for 21 of 28 patients. Five interventions were performed due to atrial fibrillation secondary to PMR. Surgery was conducted for 2 patients who developed signs of LV dysfunction and significant LA dilatation. A repair of the posterior leaflet was the most common procedure, conducted in 17 (61%) interventions. One patient had a repair of the anterior leaflet. Three (11%) surgeries included repair of both posterior and anterior leaflet. Biological valve replacement was performed in 7 (25%) surgical procedures. No patient had a transcatheter mitral valve intervention. Two deaths were registered during follow-up. One patient developed a progressive dementia. NYHA functional class 1 and normal NT-proBNP levels indicated no onset of heart failure until death. In our analysis this patient was censored. One patient was rejected valve surgery due to malignant disease, thus statistically counted as a cardiac event. There were no deaths among the operated patients.

In univariate outcome analysis, impaired peak VO_2_ defined according to the reference values proposed by Gläser et al. [18], did not predict surgical treatment of the mitral valve (Table 4). This finding remained consistent when peak VO₂ was assessed using either the Weber Classification [8] or reference values proposed by Wasserman et al. [23]. In contrast, left ventricular remodelling and the severity of PMR, together with impaired ventilatory efficiency – reflected by increased VE/VCO₂ ratios and an elevated VE/VCO₂ slope – were predictive of subsequent mitral valve surgery. We evaluated the prognostic performance of the Ventilatory Classification System [9], using a VE/VCO₂ slope cut-off ≥ 30; however, this fixed threshold did not predict the need for mitral valve surgery in our cohort. Changes in CPET indices over 1-year (CPET Δ-values) had no predictive value, neither as continuous data (S2 Table B) nor in dichotomized analysis (negative versus positive Δ-value).

**Table 4:** Outcome analysis. Follow-up 4.5 ± 2.8 years. Outcome was mitral valve surgery during follow-up.

Clinical data	hazard ratio (95% CI)	*p*
Age (years)	0.98 (0.95-1.02)	0.37
Gender (female)	1.97 (0.59-6.58)	0.27
BMI (kg/m^2^)	1.01 (0.89-1.14)	0.94
BSA (m^2^)	1.06 (0.13-8.99)	0.95
Hypertension	1.05 (0.50-2.21)	0.89
NT-pro-BNP (ng/L)	1.02 (0.99-1.05)	0.18
**Echocardiography**		
LVESD (mm)	1.11 (1.01-1.23)	**0.03**
LVEDD (mm)	0.98 (0.91-1.06)	0.63
LVEDV (mL/m^2^)	1.03 (1.01-1.05)	**0.001**
LAVI (mL/m^2^)	1.01 (1.00-1.03)	**0.047**
EROA (cm^2^)*	2.67 (1.20-5.95)	**0.02**
LVEF (≤ 60%)	1.22 (0.29-3.49)	0.74
LAVI (≥ 60mL/m^2^)	1.90 (0.55-4.94)	0.24
LVESD (≥ 40 mm)	2.04 (0.48-5.89)	0.25
**CPET**		***p***
SBP (mmHg)	1.01 (0.99-1.03)	0.19
HR (% of est max HR)	0.99 (0.95-1.03)	0.66
Watts (% of predictive vale)	1.01 (0.98-1.04)	0.59
RER	0.98 (0.94-1.02)	0.30
**Oxygen consumption**		
VO_2_ @peak (impaired; < LLN, Gläser et al.)	0.83 (0.35-1.96)	0.67
VO_2_ @peak (impaired; < 20 ml/min/kg)	0.77 (0.35-1.69)	0.51
VO_2_ @peak (impaired; < 16 ml/min/kg)	1.23 (0.20-4.14)	0.78
VO_2_ @peak (impaired; < 80% of predictive value, Wasserman et al.)	1.28 (0.61-2.66)	0.52
**O**_**2**_ **-pulse**		
O_2_-pulse (ml/beat)	1.05 (0.93-1.18)	0.42
O_2_-pulse (impaired; < LLN, Gläser et al.)	0.93 (0.35-2.46)	0.89
**Ventilatory efficiency**		
VE/VCO_2_ @AT* (>ULN, Gläser et al.)	3.10 (1.34-7.17)	**0.008**
VE/VCO_2_ @lowest value (>ULN, Sun et al.)	2.95 (1.09-8.02)	**0.03**
VE/CO_2_ slope* (>ULN, Gläser et al.)	3.17 (1.17-8.60)	**0.02**
VE/CO_2_ slope* (≥ 30)	1.96 (0.83-4.)	0.11
**Cardiac limitation**	0.72 (0.25-2.09)	0.55

Univariate Cox proportional hazards analysis was performed for clinical, echocardiographic, and cardiopulmonary exercise testing (CPET) variables. Hazard ratios represent the risk increment per unit increase in each parameter, calculated on an annual basis. Number of observations: 48 or 44

(*), as indicated. CPET and echocardiographic parameters were evaluated by comparing abnormal versus normal results, based on the cut-off values specified in the table.

Abbreviations: BMI, body mass index; BSA, body surface area; LVESD, left ventricular end-systolic volume; LVEDD, left ventricular end-diastolic volume; LVEDV, left ventricular end-diastolic volume; LAVI, left atrial volume index; EROA, effective regurgitant orifice area; LVEF, left ventricular ejection fraction; SBP, systolic blood pressure; HR, heart rate; RER, respiratory exchange ratio; VO₂, oxygen consumption; AT, anaerobic threshold; VE, ventilation; VCO₂, carbon dioxide production; LLN, lower limit of normal; ULN, upper limit of normal.

**Fig 1:**
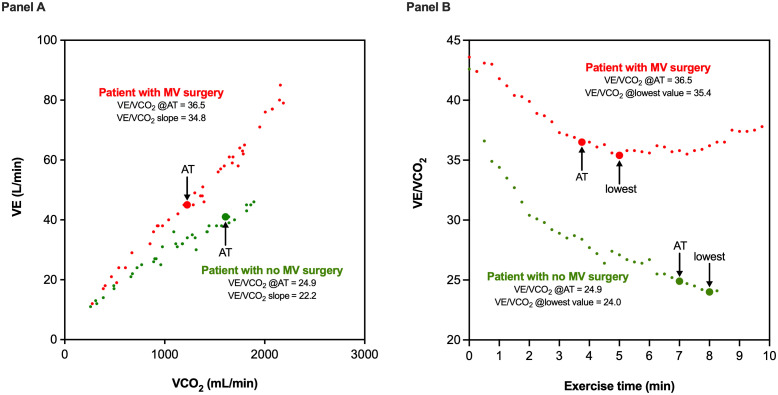
Illustrative example for a patient with (red), and a patient without (green), mitral valve surgery during follow-up. Panel A demonstrates the relationship of VE to VCO_2_ during incremental exercise. Panel B illustrates the relationship of VE/VCO_2_ to exercise time, for the same two patients. The patient with later mitral valve surgery (red) exhibits impaired ventilatory efficiency identified by: elevated VE/VCO_2_ slope (34.8 = 130% of predicted value), increased VE/VCO_2_ ratios at the anaerobic threshold (36.5 = 120% of predicted value) and at its lowest level (35,4 = 123% of predicted value). The patient without later mitral valve surgery (green) demonstrated normal ventilatory efficiency. Not illustrated in panels, both patients exhibited normal peak VO_2_ (according to normal values by Gläser et al., Wasserman et al. and Weber et al.) and normal O_2_-pulse. MV, mitral valve; VE, ventilation; VCO_2_, carbon dioxide production.

**Fig 2:**
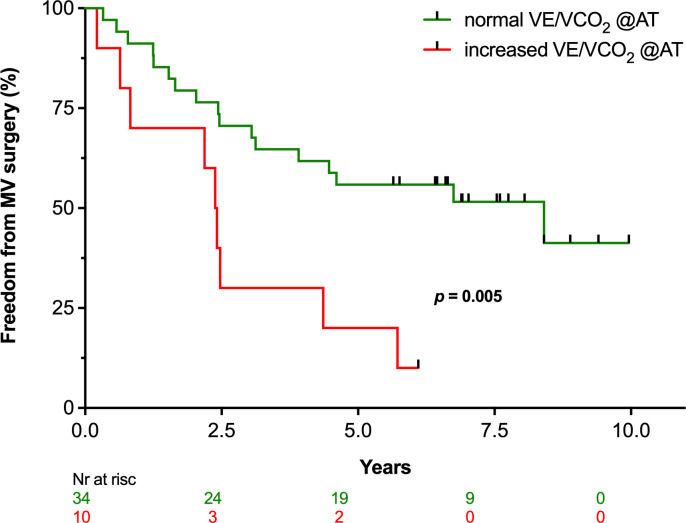
Kaplan-Meier survival curves for patients with primary mitral regurgitation, with normal (blue) and increased (red) VE/VCO_2_ ratio at the anaerobic threshold. Freedom from mitral valve surgery was decreased for patients with increased VE/VCO_2_ at the anaerobic threshold (log-rank, p = 0.005). MV, mitral valve; VE, ventilation; VCO₂, carbon dioxide production.

In exploratory multivariate models, the three ventilatory efficiency variables that were significant in univariate analysis did not demonstrate independent predictive value. Fig 1 demonstrates the relationship of VE to VCO_2_ during incremental exercise, and the relationship of VE/VCO_2_ to exercise time, in two patients with normal and impaired ventilatory efficiency, respectively. Increased VE/VCO₂ ratio at the anaerobic threshold exhibited the strongest predictive value in multivariate models. This association remained significant after adjustment for age, sex, and impaired peak VO₂ (HR 4.42 (1.52–12.92), p = 0.007), and when adjusted for echocardiographic thresholds for LV remodelling that meet current guideline-based indications for intervention (HR 3.72 (1.41–9.82), p = 0.008) (Table 5). Fig 2 demonstrates time-related incidence of valve surgery as Kaplan-Meier curves for patients with normal versus high VE/VCO₂ ratio at the anaerobic threshold, and compared with log-rank test.

**Table 5:** Exploratory multivariate Cox proportional hazards models assessing the predictive value of increased VE/VCO₂ ratio at the anaerobic threshold, for mitral valve surgery during follow-up.

	Model 1		Model 2	
	**hazard ratio (95% CI)**	***p***	**hazard ratio (95% CI)**	***p***
VE/VCO_2_ @AT (increased; > ULN, Gläser et al.)	4.42 (1.52-12.92)	**0.007**	3.72 (1.41-9.82)	**0.008**
Age (years)	0.97 (0.93-1.02)	0.24		
Gender (female)	1.91 (0.51-7.19)	0.34		
VO_2_ @peak (impaired; < 16 ml/min/kg)	2.99 (0.42-21.11)	0.27		
VO_2_ @peak (impaired; < 80% of predictive value, Wasserman et al.)	0.94 (0.34-2.60)	0.90		
VO_2_ @peak (impaired; < LLN, Gläser et al.)	0.34 (0.08-1.46)	0.15		
LAVI (≥60 mL/m2)			1.15 (0.32-4.13)	0.82
LVESD (≥ 40 mm)			0.58 (0.12-2.96)	0.52
LVEF (≤ 60%)			1.67 (0.48-5.83)	0.42

Adjustments for outcome parameters: age, gender, peak VO or echocardiographic thresholds that meet current guideline-based indications for intervention. P-values less than 0.05 were defined as significant. Numbers of observations: 44 (both models). CPET and echocardiographic parameters were evaluated by comparing abnormal versus normal results, based on the cut-off values specified in the table.

Abbreviations: VE, ventilation; VCO_2_, carbon dioxide production; AT, anaerobic threshold; VO_2_, oxygen consumption; LAVI, left atrial volume index; LVESD, left ventricular end-systolic volume; LVEF, left ventricular ejection fraction; LLN, lower limit of normal; ULN, upper limit of normal.

## Discussion

In this study, we found that impaired ventilatory efficiency predicted the need for mitral valve surgery in asymptomatic patients with severe PMR. This association was independent of reduced peak VO₂ or echocardiographic thresholds for left ventricular and atrial remodelling, as defined by current guideline-based recommendation for intervention. Among the ventilatory efficiency parameters, a pathologically increased VE/VCO₂ ratio at the anaerobic threshold demonstrated the strongest predictive value. However, both an elevated VE/VCO₂ slope and a high VE/VCO₂ ratio at its lowest value also independently predicted the need for surgery following similar adjustments.

**Fig 3:**
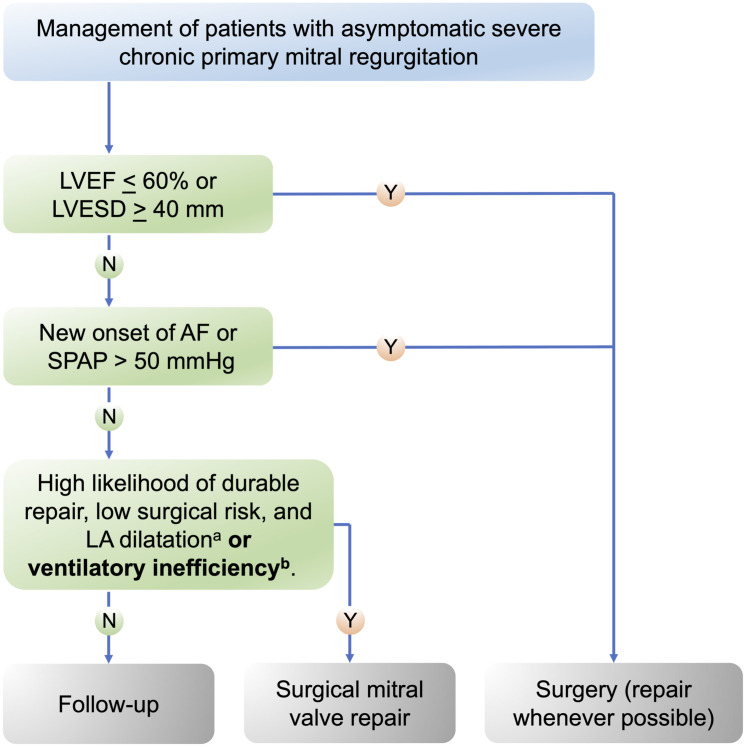
Proposed integration of ventilatory efficiency into the clinical decision-making algorithm for primary mitral regurgitation, adapted from the 2021 ESC Guidelines [1]. LVEF, left ventricular ejection fraction; LVESD, left ventricular end-systolic diameter; AF, atrial fibrillation; SPAP, systolic pulmonary arterial pressure; LA, left atrium; Y, Yes; N, No; ^a^LA dilatation defined as a volume index >_60 mL/m^2^ or diameter >_55 mm at sinus rhythm; ^b^Ventilatory inefficiency defined as an elevated VE/VCO₂ ratio or VE/VCO₂ slope, exceeding the upper limit of normal, based on appropriate age- and sex-specific reference values.

Two previous studies investigating CPET in asymptomatic patients with primary MR reported that impaired peak VO₂, based on reference values from Wasserman et al., was predictive of the need for mitral valve surgery. The study by Messika-Zeitoun et al. reported VE/VCO₂ slope values but did not assess their prognostic significance for surgical intervention [24], while the study by Coisne et al. did not include any ventilatory efficiency parameters [25]. Thus, the present study provides novel evidence supporting ventilatory efficiency as a prognostic marker, which – if validated in larger cohorts – may be suitable for integration into clinical decision-making algorithms, as proposed in Fig 3.

Impaired O2-pulse has been proposed to indicate exercise induced heart failure. However, it may not necessarily reflect stroke volume limitation, and may be altered due to impaired oxygen extraction of pulmonary causes [20]. In this study, moderate or severe pulmonary hypertension and symptomatic lung disease were exclusion criteria, arguably reducing the likelihood of O_2_-pulse being affected by pulmonary causes. VO_2_-pulse was normal in 39 (81%) patients and, similar to peak VO_2_, no predictive value was identified.

In our cohort, well-established static cut-offs, as defined by the Weber [8] and Ventilatory Classification System [9], were inferior to age- and sex-specific reference values (18,19). Nevertheless, fixed cut-offs remain appealing due to their simplicity and ease of implementation. In this cohort, the upper limits of normal for the VE/VCO₂ slope and the VE/VCO₂ ratio at the anaerobic threshold, as defined by Gläser et al., were 32.7 ± 1.8 and 33.1 ± 2.1, respectively. However, these thresholds are dependent on the characteristics of this specific cohort. In our opinion, the application of age- and sex-specific reference values offers more accurate risk stratification than static cut-off values in this clinical context and is, in our experience, feasible to implement in routine practice.

**Fig 4:**
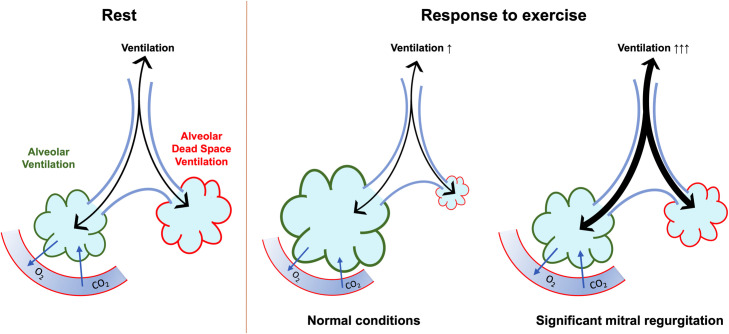
Proposed mechanisms for elevated VE/VCO₂ in patients with primary mitral regurgitation. Left illustration: Schematic representation of ventilation–perfusion distribution at rest. Total ventilation (black) denotes the cumulative rate of air entering and exiting the pulmonary system. Alveolar ventilation (green) corresponds to the fraction of ventilation reaching alveolar units that are both ventilated and perfused, thereby contributing effectively to gas exchange. In contrast, alveolar dead space (red) represents ventilated alveoli that are not perfused and thus do not participate in gas exchange. Middle illustration: Depiction of the physiological response to exercise under normal conditions without mitral regurgitation. An increase in cardiac output facilitates enhanced pulmonary perfusion, promoting more uniform ventilation–perfusion matching. This adaptation supports efficient gas exchange and maintains a normal ventilatory equivalent for carbon dioxide (VE/VCO₂). Right illustration: Illustration of an abnormal physiological response to exercise. In this setting, inadequate perfusion of ventilated alveolar units leads to a rise in alveolar dead space. To preserve effective gas exchange, minute ventilation must increase, resulting in an elevated VE/VCO₂. This maladaptive response may indicate impaired cardiac output and aligns with our hypothesis that exercise-induced worsening of mitral regurgitation contributes to perfusion deficits and the elevated VE/VCO₂ observed in the study cohort.

An increased VE/VCO_2_ ratio may occur due to low PaCO_2_ or an abnormally high alveolar dead space ventilation. A study on patients with congestive heart failure report small PaCO_2_ changes during exercise [26], for this specific patient group, ventilatory inefficiency has been proposed being due to abnormally high alveolar dead space ventilation, reflecting impaired cardiac output during exercise [6]. Pathophysiology and mechanisms of ventilatory inefficiency in PMR has to our knowledge not been discussed in detail in previous reports. The severity of an PMR may be load dependent, potentially increasing in exercise testing [27,28]. We speculate that impaired ventilatory efficiency in PMR may reflect exercise-induced increase in PMR, resulting in impaired cardiac output response to exercise. Impaired cardiac output causes poor perfusion of ventilated alveoli and abnormally high alveolar dead space ventilation (Fig 4). Future research needs to determine whether impaired ventilatory efficiency could be a signpost for exercise-induced increase in PMR.

### Strengths and limitations

All patients were followed for the primary outcome, and participation in the 1-year follow-up CPET was relatively high, with 43 out of 48 patients completing the assessment. While our data demonstrate prognostic value for CPET, they do not support the routine use of serial CPET to assess CPET Δ-values for surveillance in non-operated patients within this clinical context. Although it would have been of interest to assess all patients undergoing intervention with postoperative CPET, this was beyond the scope of the study design. Furthermore, this study does not investigate the potential predictive value of CPET in moderate PMR. It should also be noted that the cohort size was limited, and further research is needed to validate the outcome analyses presented in this study.

There are no specific reference values for CPET performed in semi-supine position and therefore we applied reference values from Gläser et al. that were obtained from cycle ergometer in upright position. A previous study reported a 6% reduction in peak VO_2_ in semi-supine, compared to upright position, in young healthy volunteers [29]. Arguably, the semi-supine position in this study had an acceptably small impact on obtained peak VO_2_.

Based on patient self-reported level of effort, RER and peak VO_2_, our cohort performed at reasonable levels of workload. For individual patients, however, uncertainty remains as to whether peak VO_2_ was recorded at adequate peak exercise; if not, the value would theoretically be falsely low. In contrast to peak VO_2_, ventilatory efficiency is assessed from data obtained from submaximal exercise phase, avoiding uncertainty whether an exercise testing was at peak or not.

V-slope, ventilatory equivalents for O_2_, end-tidal pO_2_ and RER are frequently suggested methods for determining the anaerobic threshold. Studies have however shown considerable inter-observer variability in this determination [30], and no consensus exists regarding which method is universally most consistent [20]. We chose to use the RER method in order to diminish the methodological variation in the study, however, using the RER method has arguably led to overestimated VO_2_ values at the anaerobic threshold. The overestimation is systematic, notably VO_2_ values at the anaerobic threshold exhibit no difference for operated and non-operated patients, respectively (Table 3).

The VE/VCO_2_ slope was not calculated with linear regression, as conducted in applied normal values [18], and instead determined by measurements at rest and AT. The methodological difference is arguably not significant, moreover ventilatory efficiency was also assessed by two VE/VCO_2_ ratios, and results are consistent for ventilatory efficiency predicting valve surgery.

## Conclusion

Impaired ventilatory efficiency predicted surgical treatment of the mitral valve in patients with asymptomatic severe primary mitral regurgitation, independently from echocardiographic variables. Ventilatory efficiency assessment is arguably more robust than peak VO_2_, not depending on peak exercise performance, and appears useful for risk stratification in selected patients.

## Supporting information

S1 Table A CPET Δ-values (one-year follow-up minus baseline) for patients with or without later mitral valve surgery during follow-up. (DOCX)

S2 Table B Results of univariate Cox proportional hazard analysis of CPET Δ-values. (DOCX)

## Abbrevviations

CPET: CardioPulmonary Exercise Testing
FEV_1_: Forced Expiratory Volume in 1 second
LA: Left Atrium
LV: Left Ventricle
LVEDV: Left Ventricular End-Diastolic Volume
LVEF: Left Ventricular Ejection Fraction
PMR: Primary Mitral Regurgitation
RER: Respiratory Exchange Ratio
RVSP: Right Ventricular Systolic Pressure
VCO_2_: Carbon dioxide production
VE: Ventilation
VO_2_: Oxygen consumption.
