# Supplementary material for: A prospective study on prognostic value of ventilatory efficiency in asymptomatic patients with severe primary mitral regurgitation
Source: PLoS One. 2025 Jul 9;20(7):e0326418. doi: 10.1371/journal.pone.0326418 (PMC12240333; doi:10.1371/journal.pone.0326418)
Supplement: S1 Table A — (DOCX) [file pone.0326418.s001.docx]

**Supplemental material**

| **S1. Table A.**  CPET Δ-values (one-year follow-up minus baseline) for patients with or without later mitral valve surgery during follow-up. | | | | |
| --- | --- | --- | --- | --- |
|  | **total (n=38)** | **no MV surgery (n=19)** | **MV surgery (n=19)** | ***p*** |
| Δ Watts | +1.6 ± 18.8 | -1.1 ± 20 | +4.2 ± 18 | 0.26 |
| Δ RER | -0.03 ± 0.10 | -0.02 ± 0.11 | -0.03 ± 0.09 | 0.70 |
| **Oxygen consumption** |  |  |  |  |
| Δ VO_2_ @peak (mL/min/kg) | -0.63 ± 2.6 | -0.17 ± 2.8 | -1.1 ± 2.3 | 0.37 |
| Δ VO_2_ @AT (mL/min/kg) | +1.1 ± 3.7 | +1.9 ± 4.0 | +0.3 ± 3.4 | 0.65 |
| **O_2_-pulse** |  |  |  |  |
| Δ O_2_-pulse (mL/min/bpm) | +0.8 ± 2.5 | +1.4 ± 2.1 | +0.3 ± 2.8 | 0.23 |
| **Ventilatory efficiency** |  |  |  |  |
| Δ VE/VCO_2_ @AT | +0.7 ± 1.8 | +0.2 ± 1.8 | +1.3 ± 1.6 | 0.10 |
| Δ VE/VCO_2_ @lowest value | +0.4 ± 1.7 | +0.2 ± 2.0 | +0.6 ± 1.5 | 0.42 |
| Δ VE/CO_2_ slope | +0.9 ± 2.2 | +0.2 ± 2.2 | +1.6 ± 2.1 | 0.57 |
| CPET, cardiopulmonary exercise testing; MV, mitral valve; RER, respiratory exchange ratio; VO_2_, oxygen consumption; AT, anaerobic threshold; VE, ventilation; VCO_2_, carbon dioxide production. | | | | |
