# Supplementary material for: A prospective study on prognostic value of ventilatory efficiency in asymptomatic patients with severe primary mitral regurgitation
Source: PLoS One. 2025 Jul 9;20(7):e0326418. doi: 10.1371/journal.pone.0326418 (PMC12240333; doi:10.1371/journal.pone.0326418)
Supplement: S2 Table B — (DOCX) [file pone.0326418.s002.docx]

**Supplemental material**

| **S2. Table B.**  Results of univariate Cox proportional hazard analysis of CPET Δ-values. | | |
| --- | --- | --- |
|  | **hazard ratio (95% int)** | ***p*** |
| Δ Watts | 1.01 (0.99-1.04) | 0.27 |
| Δ RER | 0.45 (0.00-124.3) | 0.78 |
| Δ VO_2_ @peak (mL/min/kg) * | 0.99 (0.97-1.01) | 0.34 |
| Δ VO_2_ @AT (mL/min/kg) * | 0.99 (0.96-1.01) | 0.31 |
| Δ O_2_-pulse (mL/min/bpm) | 0.98 (0.75-1.29) | 0.91 |
| Δ VE/VCO_2_ @AT | 1.29 (0.95-1.75) | 0.10 |
| Δ VE/VCO_2_ @lowest value | 1.01 (0.55-1.83) | 0.98 |
| Δ VE/VCO_2_ slope | 1.16 (0.91-1.47) | 0.24 |
| Hazard ratio increments per year as per parameter unit, or per 10 units (*). Numbers of observations: 38 (Δ watts, Δ VO_2_peak_, ΔO_2_-pulse), 26 (Δ RER), 24 (Δ VE/VCO_2_ slope), 21 (Δ VO_2_AT_, Δ VE/VCO_2_AT_), 18 (ΔVE/VCO_2_lowest_). CPET, cardiopulmonary exercise testing; RER, respiratory exchange ratio; VO_2_, oxygen consumption; AT, anaerobic threshold; VE, ventilation; VCO_2_, carbon dioxide production. | | |
